# *Salmonella* Serovars from Humans and Other Sources in Thailand, 1993–2002

**DOI:** 10.3201/eid1001.02-0781

**Published:** 2004-01

**Authors:** Aroon Bangtrakulnonth, Srirat Pornreongwong, Chaiwat Pulsrikarn, Pathom Sawanpanyalert, Rene S. Hendriksen, Danilo M. A. Lo Fo Wong, Frank M. Aarestrup

**Affiliations:** * World Health Organization National Salmonella and Shigella Centre, Bangkok, Thailand; † WHO Collaborating Center for Antimicrobial Resistance in Foodborne Pathogens, Copenhagen, Denmark

## Abstract

We serotyped 44,087 *Salmonella* isolates from humans and 26,148 from other sources from 1993 through 2002. The most common serovar causing human salmonellosis in Thailand was *Salmonella enterica* Weltevreden. Serovars causing human infections in Thailand differ from those in other countries and seem to be related to *Salmonella* serovars in different food products and reservoirs.

*Salmonella enterica* is one of the most common causes of human gastroenteritis. The infection is caused primarily by improper handling and digestion of uncooked food; a large number of food animal sources have been identified as reservoirs of the bacteria (*1*). More than 2,500 serovars of *S. enterica* have been identified; most have been described as the cause of human infections, but only a limited number of serovars are of public health importance. *S. enteric*a serovars Typhimurium and Enteritidis have been reported to be the most common causes of human salmonellosis (*1*,*2*). However, in some regions other serovars are of greater importance (*3*,*4*).

Different serovars in one country can be of global importance because of travel and animal and food product trade. Knowledge about the occurrence and epidemiology of different serovars in different countries and geographic regions may assist in the recognition and tracing of new emerging pathogens. We review the trends in serovars of *Salmonella* causing infections in humans and potential reservoirs in Thailand during 1993 to 2002.

## The Study

The World Health Organization (WHO) National Salmonella and Shigella Centre in Bangkok receives all isolates suspected to be *Salmonella* from all diagnostic laboratories throughout Thailand. From1993 to 2002, these have included 62 governmental general hospitals; 5 private hospitals; 12 regional medical centers; 3 laboratories within the Department of Livestock Development; 6 laboratories within the Fisheries Department, the Laboratory of Bangkok Health Center, and U.S. Embassy; and 28 food industry laboratories. All laboratories are encouraged to use both direct plating and enrichment broth to isolate *Salmonella*. For enrichment the laboratories use Selenite, Tetrathionate or Rappaport-Vassiliadis Soya Peptone broth. For direct plating they use Salmonella/Shigella, Xylose Lysine Desoxycholate, Brilliant Green or Modified Semi-solid Rappaport-Vassiliadis agar. Accepted brands are Difco, Oxoid, MAST, BBL, and Merck. For shipment the isolates are stabbed in nutrient agar sticks.

On arrival, all isolates were purified and confirmed to be *Salmonella* on the basis of reactions on triple sugar iron agar and lysine indol motility agar. All strains identified as *S. enterica* were serotyped according to the Kauffman-White serotyping scheme (*5*). *Salmonella* antisera (S & A Reagent Laboratory LMT, Bangkok, Thailand) were used for serotyping. From 1993 through 2002, a total of 70,235 isolates received were confirmed as *S. enterica* and serotyped.

**Figure 1:**
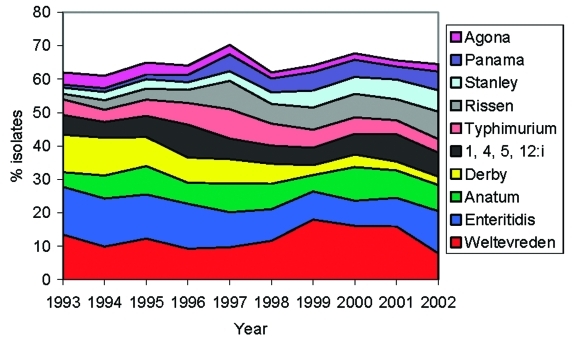
Trends over time for the 10 most common *Salmonella* serovars causing infections in humans between 1993 and 2002.

A total of 118 serovars were identified among the 44,087 isolates from humans. The 25 most common serovars accounted for 86% of the isolates, the 10 most common for 64.7%, and the 5 most common serovars (*S*. Weltevreden, *S*. Enteritidis, *S*. Anatum, *S*. Derby, *S*. 1,4,5,12:i) for 44.3% of the isolates (Table 1). The proportion of *S*. Weltevreden isolates decreased from 13.5% in 1993 to 9.3% in 1996 and has since increased to 18% in 1999, 15.9% in 2001, and 7.9% in 2002. The proportion of *S*. Enteritidis isolates has decreased during the period from 14% to 9% in 2001 and 12.6% in 2002. The proportion of *S*. Anatum has varied from 4% to 10%. *Salmonella* (1,4,5,12:i) peaked in 1996 at 10% but has otherwise been 6%-8%. The proportion of *S*. Typhimurium isolates peaked in 1997 at 9%, but was 4% in 2002. An increase has been observed for *S*. Rissen (2% to 8%), *S*. Stanley (2% to 6%), *S*. Panama (1% to 6%), and *S*. Schwarzengrund (0% to 2%), while a decrease has been observed for *S*. Derby (11% to 3%) and *S*. Krefeld (4.5% to 1%). The trends of the most common serovars are shown in Figure 1.

**Table 1:** The 25 most common serovars of *Salmonella* isolates from humans reported annually, 1993–2002, Thailand

Serovar	Y and no. of isolates (%)											Total
	1993	1994	1995	1996	1997	1998		1999	2000	2001	2002	
Weltevreden	443 (13.5)	574 (9.9)	816 (12.3)	337 (9.3)	335 (9.7)	485 (11.6)		862 (18.0)	660 (16.1)	657 (15.9)	322 (7.9)	5,491 (12.5)
Enteritidis	471 (14.3)	833 (14.4)	877 (13.2)	489 (13.4)	365 (10.5)		396 (9.5)	401 (8.4)	306 (7.5)	357 (8.6)	515 (12.6)	5,010 (11.4)
Anatum	146 (4.4)	397 (6.9)	568 (8.5)	229 (6.3)	298 (8.6)		320 (7.6)	235 (4.9)	412 (10.1)	340 (8.2)	318 (7.8)	3,263 (7.4)
Derby	368 (11.2)	650 (11.3)	576 (8.7)	277 (7.6)	252 (7.3)		251 (6.0)	141 (3.0)	156 (3.8)	111 (2.7)	107 (2.6)	2,889 (6.6)
1, 4, 5, 12:i:-ssp.I	193 (5.9)	272 (4.7)	422 (6.3)	355 (9.8)	212 (6.1)		228 (5.4)	248 (5.2)	248 (6.1)	336 (8.1)	290 (7.1)	2,804 (6.4)
Typhimurium	154 (4.7)	216 (3.7)	326 (4.9)	238 (6.5)	305 (8.8)		278 (6.6)	258 (5.4)	205 (5.0)	175 (4.2)	167 (4.1)	2,322 (5.3)
Rissen	54 (1.6)	162 (2.8)	222 (3.3)	143 (3.9)	295 (8.5)		246 (5.9)	317 (6.6)	287 (7.0)	259 (6.3)	334 (8.2)	2,319 (5.3)
Stanley	64 (1.9)	147 (2.5)	186 (2.8)	85 (2.3)	99 (2.9)		147 (3.5)	245 (5.1)	210 (5.1)	242 (5.9)	263 (6.4)	1,688 (3.8)
Panama	31 (0.9)	64 (1.1)	9 (1.4)	80 (2.2)	173 (5.0)		172 (4.1)	264 (5.5)	209 (5.1)	160 (3.9)	230 (5.6)	1,474 (3.3)
Agona	118 (3.6)	215 (3.7)	236 (3.6)	103 (2.8)	102 (2.9)		76 (1.8)	95 (2.0)	76 (1.9)	75 (1.8)	90 (2.2)	1,096 (2.7)
Choleraesuis	99 (3.0)	87 (1.5)	139 (2.1)	122 (3.4)	68 (2.0)		118 (2.8)	92 (1.9)	69 (1.7)	85 (2.1)	186 (4.5)	1,065 (2.4)
Hadar	64 (1.9)	8 (1.4)	198 (3.0)	67 (1.8)	80 (2.3)		8 (2.0)	96 (2.0)	106 (2.6)	136 (3.3)	112 (2.7)	1,023 (2.3)
Paratyphi A	76 (2.3)	107 (1.9)	134 (2.0)	330 (9.1)	47 (1.4)		157 (3.8)	108 (2.3)	—	15 (0.4)	7 (1.7)	981 (2.2)
Krefeld	149 (4.5)	129 (2.2)	135 (2.0)	52 (1.4)	74 (2.1)		67 (1.6)	72 (1.5)	36 (0.9)	32 (0.8)	39 (1.0)	785 (1.8)
Paratyphi B var Java	31 (0.9)	40 (0.7)	66 (1.0)	46 (1.3)	61 (1.8)		56 (1.3)	113 (2.4)	120 (2.9)	117 (2.8)	48 (1.2)	698 (1.6)
Typhi	61 (1.9)	53 (0.9)	41 (0.6)	42 (1.2)	43 (1.2)		64 (1.5)	68 (1.4)	—	213 (5.2)	82 (2.0)	667 (1.5)
Virchow	52 (1.6)	69 (1.2)	77 (1.2)	28 (0.7)	35 (1.0)		45 (1.1)	89 (1.9)	70 (1.7)	102 (2.5)	79 (1.9)	646 (1.5)
Lexington	40 (1.2)	67 (1.2)	66 (1.0)	35 (1.0)	45 (1.3)		60 (1.4)	68 (1.4)	56 (1.4)	88 (2.1)	52 (1.3)	577 (1.3)
Blockley	82 (2.5)	78 (1.4)	53 (0.8)	27 (0.7)	20 (0.6)		49 (1.2)	45 (0.9)	56 (1.4)	47 (1.1)	41 (1.0)	498 (1.1)
Hvittingfoss	12 (0.4)	94 (1.6)	125 (1.9)	27 (0.7)	12 (0.3)		16 (0.4)	66 (1.4)	41 (1.0)	33 (0.8)	35 (0.9)	461 (1.0)
Senftenberg	62 (1.9)	126 (2.2)	64 (1.0)	16 (0.4)	28 (0.8)		37 (0.9)	29 (0.6)	20 (0.5)	26 (0.6)	44 (1.1)	452 (1.0)
Bovismorbificans	32 (1.0)	54 (0.9)	87 (1.3)	16 (0.4)	37 (1.1)		42 (1.0)	56 (1.2)	30 (0.7)	29 (0.7)	56 (1.4)	439 (1.0)
London	27 (0.8)	92 (1.6)	72 (1.1)	45 (1.2)	67 (1.9)		71 (1.7)	24 (0.5)	15 (0.4)	8 (0.2)	0 (0.0)	421 (1.0)
Schwarzengrund	0 (0.0)	9 (0.2)	3 (0.0)	3 (0.1)	6 (0.2)		26 (0.6)	76 (1.6)	99 (2.4)	98 (2.4)	52 (1.3)	372 (0.8)
Emek	31 (0.9)	38 (0.7)	56 (0.8)	29 (0.8)	29 (0.8)		51 (1.2)	30 (0.6)	26 (0.7)	27 (0.7)	30 (0.7)	347 (0.8)
Other	424 (12.9)	1,116 (19.3)	1,011 (15.2)	415 (11.4)	380 (11.0)		643 (15.4)	679 (14.2)	577 (14.1)	366 (8.9)	598 (14.6)	6,299 (14.3)
Total	3,284	5,770	6,647	3,636	3,468		4,184	4,777	4,090	4,134	4,097	44,087

**Figure 2:**
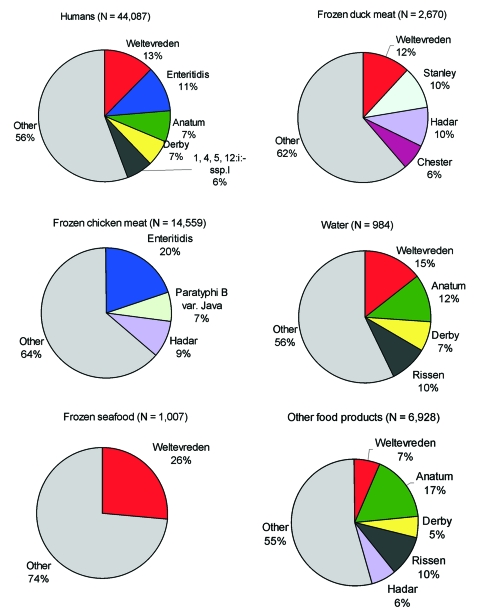
Distribution of the most common *Salmonella* serovars among the different reservoirs. Only serovars accounting for more than 6% of the isolates were included.

Samples have not been systematically taken from the different reservoirs for *Salmonella* infections in humans. However, data from samples were available from chicken, seafood, other food products, and water for all 10 years. Data about duck were only available from 1998 to 2002. The 10 most common serovars from all sources are given in Table 2. All serovars that were represented with >6% of the isolates are given in Figure 2.

**Table 2:** Distribution of the 10 most common serovars from the different reservoirs, Thailand^a^

Serovar	Reservoir and no. of isolates (%)					
	Humans	Frozen chicken	Frozen seafood	Frozen duck	Other food products	Water
Weltevreden	5,491 (12.5)	**—**	265 (26.3)	320 (12.0)	457 (6.6)	143 (14.5)
Enteritidis	5,010 (11.4)	2,901 (19.9)	14 (1.4)	—	309 (4.5)	22 (2.2)
Anatum	3,263 (7.4)	423 (2.9)	20 (2.0)	—	1,177 (17.0)	113 (11.5)
Derby	2,889 (6.6)	—	20 (2.0)	—	370 (5.3)	71 (7.2)
1, 4, 5, 12:i:-ssp.I	2,804 (6.4)	—	—	—	—	—
Typhimurium	2,322 (5.3)	—	12 (1.2)	—	198 (2.9)	—
Rissen	2,319 (5.3)	—	21 (2.1)	—	712 (10.3)	93 (9.5)
Stanley	1,688 (3.8)	—	20 (2.0)	279 (10.4)	—	—
Panama	1,474 (3.3)	—	—	41 (1.5)	254 (3.7)	47 (4.8)
Agona	1,096 (2.7)	452 (3.1)	—	80 (3.0)	273 (3.9)	39 (4.0)
Paratyphi B var Java	—	1037 (7.1)	—	—	—	—
Hadar	—	1,357 (9.3)	21 (2.1)	263 (9.9)	439 (6.3)	—
Virchow	—	863 (5.9)	—	—	249 (3.6)	27 (2.7)
Schwarzengrund	—	565 (3.9)	—	—	—	—
Emek	—	359 (2.5)	—	—	—	—
Blockley	—	676 (4.6)	—	—	—	—
Amsterdam	—	368 (2.5)	—	103 (3.9)	—	—
Seftenberg	—	—	49 (4.9)	86 (3.2)	—	—
Lexington	—	—	47 (4.7)	—	—	35 (3.6)
Newport	—	—	—	100 (3.7)	—	—
Tennessee	—	—	—	77 (2.9)	—	—
Chester	—	—	—	171 (6.4)	—	—
London	—	—	—	—	—	22 (2.2)
Other	15,824 (35.9)	5,558 (38.2)	518 (51.4)	1,150 (43.1)	2,490 (35.9)	372 (37.8)
Total	44,087	14,559	1,007	2,670	6,928	984

^a^—, not among the top 10 serovars.

*S*. Enteritidis (19.9%) was the most common serovar among the 14,559 *Salmonella* isolates from chicken, followed by *S*. Hadar (9.3%) and *S*. Paratyphi B var Java (7.1%). The most common serovar among the 1,007 isolates from seafood was *S*. Weltevreden (26%); among the 2,670 isolates from duck, the most commonly isolated serovars were, *S*. Weltevreden (12%), *S*. Hadar (9.9%), *S*. Stanley (10.4%), and *S*. Chester (6.4%). Among the 6,928 isolates from other food products, *S*. Anatum (17.0%), *S*. Rissen (10.3%), *S*. Hadar (6.3%), and *S*. Weltevreden (6.6%) were the predominant serovars; among the 984 isolates from water, they were *S*. Weltevreden (14.5%), *S*. Anatum (11.5%), *S*. Rissen (9.5%), and *S*. Derby (7.2%).

Similar trends were detected for some of the serovars among isolates causing infections in humans and contamination in some of the food products (Table 3 and Table 4). In chicken, the relative incidence of *S*. Enteritidis decreased from 17.1% in 1993 and 33.8% in 1994 to 6.6% in 2001 and increased to 14.2% in 2002. Similarly, S. Derby decreased from 6.7% in 1993 to 1.1% in 2002. In contrast, *S*. Schwarzengrund increased from 0.3% in 1993 to 26.2% in 2001, with a decrease to 7.2% in 2002. *S*. Rissen in other food products increased from 4.7% (1993) to 14.7% (2002); *S*. Panama was found in <3% (1993) to >4% (2002); *S*. Stanley was found in 1% (1993) to 7.3% (2002); and *S*. Schwarzengrund was found in 0% (1993) to 3% (2001), followed by a decrease to 1% (2002).

**Table 3:** Annual number of reported *Salmonella* isolates from chicken in which changes in number of infections in humans were observed

*Salmonella* serovar	Trend in human isolates (%)	% of isolates									
		Chicken									
		1993	1994	1995	1996	1997	1998	1999	2000	2001	2002
Enteritidis	14 to 13	17.1	33.8	29.5	15.0	18.5	15.3	14.2	12.0	6.6	14.2
Derby	11 to 3	6.7	0.9	2.6	4.3	1.2	0.4	1.3	1.1	1.6	1.1
Schwarzengrund	0 to 1	0.3	0.1	0.0	0.2	1.6	1.4	3.5	15.0	26.2	7.2
No. of isolates		1,909	2,370	2,010	1,005	1,534	1,414	908	952	836	1,621

**Table 4:** Annual number of reported *Salmonella* isolates from other food products in which changes in number of infections in humans were observed

*Salmonella* serovar	Trend in human isolates (%)	% of isolates									
		Other food products									
		1993	1994	1995	1996	1997	1998	1999	2000	2001	2002
Rissen	2 to 8	4.7	11.0	8.9	10.0	3.6	7.0	8.6	15.3	14.2	14.7
Panama	1 to 6	2.8	1.6	1.6	2.9	2.1	0.6	4.2	6.7	5.3	4.2
Stanley	2 to 6	0.9	1.1	1.6	1.8	0.9	1.9	3.0	1.9	1.9	7.3
Schwarzengrund	0 to 1	0.0	0.0	0.0	0.0	0.4	0.6	1.7	3.8	2.9	1.0
No. of isolates		107	182	258	450	1,498	483	999	946	697	1,308

## Conclusions

*S.* *enterica* continues to be one of the most important causes of foodborne gastrointestinal infections in humans. During the last few decades *S*. Enteritidis and *S*. Typhimurium have emerged as the two predominant serovars in most Western countries. The epidemiology of these serovars has been studied, and different programs have been established to limit the spread of these serovars. However, other serovars may have a different epidemiology, and conditions are optimal for spread between reservoirs in some countries.

Our report indicates that *S*. Weltevreden was the most common serovar isolated from humans in Thailand. A similar finding has been reported from Malaysia (*3*), and *S*. Weltevreden was the most common serovar to cause human infections in India during the early 1970s (*6*). Before 1970, this serovar constituted <4% of total human salmonellosis. The number of reported infections caused by *S*. Weltevreden increased in the early 70s; in 1972, this serovar constituted 29.1% of all *Salmonella* infections in India. Thong et al. (*7*) found the same types of *S*. Weltevreden among isolates infecting humans and those in raw vegetables, suggesting that this is a potential reservoir of this serovar in Malaysia. Raw vegetables may, however, be contaminated by both feces and water. In a recent study from the United States, *S*. Weltevreden was the most common serovar found in seafood mainly imported from Thailand and Malaysia (*8*). These observations could point to a water-related source for *S*. Weltevreden. *S*. Weltevreden was the most common serovar in isolates from seafood, water, and duck, which suggests a water-related source for this serovar in Thailand.

*S.* Enteritidis infections in humans in Thailand increased from 1.3% in 1990 to 14% in 1993 to 1994 (*4*). Thus, Thailand has also been part of the global pandemic of *S.* Enteritidis observed in the late 1980s (*9*). The 1995 global survey conducted by WHO showed that the global pandemic has continued and expanded (*2*). The *S.* Enteritidis pandemic appears to have ended in 1997; this finding is similar to the decrease observed in Thailand, where *S.* Enteritidis has decreased during the last decade. However, this serovar is still an important cause of human infections, reflected in the increase in 2002. The frequent occurrence of this serovar in chickens suggests that poultry may be an important reservoir, a finding that is consistent with almost all other studies in other countries (*10*). Eggs have also been found to be important reservoirs in other countries (*10*) but were not examined in this study.

*S.* Anatum has consistently been one of the most important causes of salmonellosis in Thailand. The main reservoirs seem to be other food products and water. This serovar has previously been isolated from a large number of different animal sources.

The importance of *S.* Derby has decreased in Thailand. *S.* Derby has been associated with pigs (*11*,*12*). Pork and other swine products were not sampled in this report, but a frequent occurrence of the serovar was observed among unspecified other food products, which could include pork.

*Salmonella* isolates of serovar (1,4,5,12:I) were frequently found in isolates from humans, but infrequently in isolates from the different food reservoirs. Thirty isolates from Thailand were examined by phage typing and susceptibility testing (data not shown). A variable resistance pattern was observed, and five (17%) had a resistance pattern and phage reaction in agreement with *S.* Typhimurium U302. Twenty (67%) did not react with any phages. Some of these isolates from Thailand might be *S.* Typhimurium, but a large number might belong to other serovars.

*S.* Typhimurium is among the most prevalent serovars in Europe and America and of growing importance in Southeast Asia, Africa, and the Western Pacific (*2*). In Thailand, the importance of this serovar has not increased and continues to account for 5% of all human infections. *S.* Typhimurium can be found among a large number of different animal reservoirs; no specific source has been found.

*S.* Rissen has been isolated infrequently as a cause of human infections, and limited information about the potential reservoirs are available. The importance of this serovar seems to be increasing in Thailand. A specific reservoir for *S.* Rissen has not been identified, but the frequent occurrence of this serovar in *Salmonella* from water and other food products indicates that a foodborne or waterborne reservoir is a possibility.

*S.* Stanley infections were among the 15 most common serovars in 12 of 104 countries (*2*) in 1995. The relative importance of this serovar seems to be increasing in Thailand, and the only reservoir where the serovar was found in high frequency was duck.

*S.* Panama has been the cause of a number of outbreaks in different countries (*13*) and among the 15 most common serovars in 10 of 104 countries in a recent WHO survey (*2*). To date, no specific reservoir has been identified. The importance of this serovar seems to be increasing in Thailand and may be correlated to a simultaneous increase among food products.

*S.* Schwarzengrund has only been isolated sporadically from infections in humans and from animal sources. The number of infections caused by this serovar in Thailand is still very low. However, the proportion seems to be increasing in isolates from humans and chicken. From 1993 to 1997, this serovar constituted <0.2% of all reported humans salmonellosis, a proportion that increased from 1% to 2% in 2001 to 2002. During the same period, the proportion among isolates from chicken has increased from a similar figure to 26% of all isolates in 2001 and 7.2% in 2002. Thus, some evidence exists that this serovar could be increasing in importance in the chicken population and subsequently in humans in Thailand. *S.* Schwarzengrund has also been found in chickens in other studies, suggesting that poultry could be the most common reservoir (*14*,*15*).

A large number of other serovars were also isolated from humans and nonhuman sources. However, some serovars most commonly found in the 1995 WHO survey were only infrequently observed in Thailand. *S.* Hadar was the 12th most common serotype and *S.* Typhi was the 16th, while other commonly observed serovars such as *S.* Infantis and *S.* Newport were not observed among the 25 most common serovars. *S.* Hadar has been associated with poultry (*15*). This finding was also observed in this study; *S.* Hadar was frequently isolated from chicken and duck. However, this finding does not seem to have a major impact on the number of infections in humans.

The results from this report show that serovars can differ largely between countries and regions, which is likely related to the available reservoirs for persistence and spread of *Salmonella* infections. The distribution of serovars causing infections in Thailand differs markedly from those reported in other countries and seems to be related to the *Salmonella* serovars in the different food products and other reservoirs for infections. Of particular interest is the frequent occurrence of *S.* Weltevreden and recent increase in occurrence of *S.* Rissen, *S.* Stanley, and *S.* Schwarzengrund.
